# The PEARLS Program: Point‐of‐Care Ultrasound for Emergency and Acute Care in Resource‐Limited Settings

**DOI:** 10.1002/ajum.70014

**Published:** 2025-07-07

**Authors:** Bilal Hafeez, Wai Chung Tse, Ivan Chan, Vincent Atua, Ignatius Junior Bolokon, Alu Kali, Sam Orde, Jo McCann, Jonathan Henry, Lewis McLean

**Affiliations:** ^1^ Faculty of Medicine, Dentistry and Health Sciences The University of Melbourne Melbourne Victoria Australia; ^2^ Austin Health Melbourne Victoria Australia; ^3^ Disease Elimination Program Burnet Institute Melbourne Victoria Australia; ^4^ Emergency Department Vila Central Hospital Port Vila Vanuatu; ^5^ Emergency Department Rabaul Provincial Hospital Rabaul Papua New Guinea; ^6^ Intensive Care Unit, Department of Anaesthesia & Intensive Care Port Moresby General Hospital Papua New Guinea; ^7^ Department of Intensive Care Medicine Nepean Hospital Sydney New South Wales Australia; ^8^ Department of General Practice and Rural Health Otago Medical School Dunedin New Zealand; ^9^ Emergency Department Peninsula Health Melbourne Victoria Australia; ^10^ Intensive Care Unit John Hunter Hospital Newcastle New South Wales Australia

**Keywords:** EFAST, FELS, POCUS for Emergency and Acute care in Resource‐Limited Settings, point‐of‐care ultrasound, remote image review

## Abstract

**Introduction:**

Point‐of‐care ultrasound (POCUS) can be very useful in low‐resource settings, where access to radiology is often limited. The PEARLS (POCUS in Emergency and Acute care in Resource‐Limited Settings) program is a scalable, innovative approach to POCUS training for clinicians in low‐ and middle‐income countries (LMICs), where access to imaging modalities is frequently limited.

**Methods:**

A review of the PEARLS program was conducted using participant data that were gathered from participants in Vanuatu, Tonga, and Papua New Guinea (PNG), tracking the number and type of scans uploaded and overall engagement with the training. The PEARLS program begins with a three‐day in‐person course focused on hands‐on practice using portable ultrasound devices. Clinicians are provided with devices and guided on how to store images securely online. After the course, each participant is paired with an experienced coach who offers ongoing remote support through regular image reviews and a series of follow‐up lectures. One year later, the program team revisits the site, and selected participants are transitioned into faculty roles to help train others in a train‐the‐trainer model.

**Results:**

Across 40 participants, a total of 576 scans were uploaded following training. The extended FAST exam was the predominant modality, followed by critical care echocardiography and first‐trimester pregnancy assessments. Although remote mentoring maintained high levels of engagement, only one participant achieved the full competency benchmark of 25 uploaded scans.

**Discussion:**

These findings illustrate that a longitudinal training model with remote mentorship can improve POCUS proficiency in LMIC settings. However, challenges such as inconsistent internet access and equipment sharing persist, potentially limiting sustained competency.

**Conclusion:**

The PEARLS program presents a scalable, sustainable framework for POCUS education in resource‐limited environments, fostering local expertise and enhancing acute care diagnostics.

**Summary**

- Point‐of‐care ultrasound (POCUS) has significant potential as an imaging modality in low‐ and middle‐income countries (LMICs).
- We introduce a comprehensive POCUS training program to doctors in LMICs called ‘PEARLS’: POCUS for Emergency and Acute care in Resource‐Limited Settings.
- Easy to use, hand‐held ultrasound devices with the ability for secure cloud platform image storage are key.
- Firstly, an in‐person course provides LMIC doctors with initial skills.
- Participants are then paired with ‘coaches’ and remote training is provided by reviewing images uploaded on the server, along with a postcourse lecture series.
- The PEARLS team returns and key participants who were trained over the previous year graduate to become faculty for the next course which enhances sustainability and maximises the chance of further learners progressing to POCUS mastery.

## 1 Introduction

Point‐of‐care ultrasound (POCUS) has significant unmet potential in resource‐limited settings, where there is typically high disease burden, limited radiology services (e.g., CT, comprehensive ultrasound and even X‐ray) and prompt requirement for medical care. POCUS training in low‐ and middle‐income countries (LMICs) is often limited by postcourse skill decay, due to both inadequate equipment access and inability to maintain and develop learners' skills [1]. We describe the PEARLS (POCUS for Emergency and Acute care in Resource‐Limited Settings) program, including several innovative features that overcome these limitations, which aim to enhance sustainability and facilitate postcourse learner development through remote image review and feedback.

POCUS is an imaging modality which is performed by the treating clinician at the bedside and can help identify significant pathology quickly and efficiently. Ultrasound's safety profile, low cost, reusability, immediate availability of results and practicality make it an ideal adjunct for clinicians in LMICs [2]. The World Health Organisation (WHO) has deemed ultrasound machines to be a significant medical technology for resource‐limited settings, but further work is needed to demonstrate how to integrate its use into these specific environments [3].

Access to all forms of radiology including ultrasound in LMICs is often severely restricted by understaffing, lack of ongoing training and ageing equipment with inadequate maintenance access [4]. A recent case series reported the radiology department of a Pacific Island regional hospital having its X‐ray machine out of service for several months [4]. Barriers to widespread POCUS uptake have included insufficient training programmes, limited in‐person training exposure, and a lack of on‐the‐ground skilled POCUS educators [2, 3]. However, longitudinal training programmes have been demonstrated to be valuable for POCUS learners [5]. Some studies have also demonstrated engagement of tele‐ultrasonography with skilled educators can supplement ongoing learning [6].

### 1.1 PEARLS‐ED (Emergency Department)

Between 2022 and 2023, a 3‐day PEARLS‐ED course was run three times—in Vanuatu, Tonga, and Papua New Guinea (PNG). The content of E‐FAST, 1st trimester US and US‐guided IV cannulation aimed to target high‐morbidity pathologies frequently diagnosed in resource‐limited settings, for which management options exist. Precourse learning included relevant online educational video modules and access to a free online medical textbook [7]. At the start of the course, learners were provided with handheld Butterfly (Butterfly Network Incorporated, Burlington, Massachusetts, USA) ultrasound transducers (Butterfly IQ+, Frequency 1‐10–MHz) and compatible smartphone or tablet devices, if not already in their possession. Where funding restrictions were present, often equipment was shared between two or more learners working at the same institution. The first two days included brief lectures reinforcing the precourse learning, with maximised hands‐on scanning time, followed by a FAST exam Objective Structured Clinical Examination (OSCE) to assess competence (Appendix 1). Other elements included case‐based discussions and case presentations. The third day consisted of supervised in‐hospital scanning, where learners began to appreciate real‐life case abnormalities. A multi choice quesstion (MCQ) test was also conducted before and after the course to track learners' information uptake; this was repeated at an interval postcourse to assess knowledge retention.

### 1.2 PEARLS‐CC (Critical Care)

The PEARLS‐CC course functions as a natural progression for prior PEARLS‐ED attendees, or as a standalone course for critical care POCUS. It is primarily targeted at emergency doctors, anaesthetists, intensivists and other acute care doctors managing critically unwell patients in emergency departments, intensive care units, high dependency units, operative theatres or elsewhere in their practice. Similarly structured to PEARLS‐ED, the content was centred on echocardiography, with the addition of lung ultrasound and procedural guidance. A Basic Critical Care Echocardiography (BCCE) OSCE was used to assess competence at the end of day 2 (Appendix 2).

### 1.3 Remote Image Review and Ongoing Mentoring

Sustainability and creating local mentors for future POCUS training are key. A small proportion of POCUS learners progress to competence, and a lack of mentors is one contributor [8]. A critical element of the PEARLS program is the opportunity for postcourse image review through several mechanisms. Learners seamlessly upload anonymised images to a secure, password‐protected online cloud as they scan for remote review and educational feedback by the volunteer course faculty. Each learner is assigned to one of the course faculty as their own PEARLS Coach, so postcourse imaging feedback can be tailored to their learning style by an educator who knows them personally. Faculty aim for their feedback to occur promptly to maximise its impact.

After uploading the required 25 studies, learners are designated as having completed the PEARLS program. After completing the program, learners can remain on the cloud and continue receiving feedback on their ongoing imaging. Other steps to optimise postcourse engagement have included an informal messaging ‘chat group’ including course participants and instructors, which has been shown to work in an African setting [9].

### 1.4 Pearls Lecture Series and Image Review Sessions

Since February 2023, a monthly online lecture series has been delivered by fifteen different world‐renowned global health experts, with recordings being uploaded online for later viewing. A free open access event, attendance is growing and currently averages 15–20 virtual attendees from around the world per lecture, including prior PEARLS course participants. A monthly image review session has also been implemented since April 2024, where learners can log on to an online video call and share a recent case, which allows for an objective review of imaging in a group discussion format. These sessions are moderated by a PEARLS faculty member and allow for peer‐to‐peer learning as well as expert review with a wider PEARLS faculty group.

## 2 Methods

This study evaluated the implementation and outcomes of the PEARLS training in Tonga and Vanuatu. The aim is to examine the frequency and scan types uploaded by participants to the Butterfly iQ cloud platform [10]. Data from the Butterfly cloud—collected up to 06/09/2024—were analysed for the following outcomes: scan frequency, scan modality, and participation in the longitudinal POCUS training.

The PEARLS training programme was conducted in three country cohorts: in Tonga, two PEARLS‐ED courses were conducted, with collected data from the 2023 session. In PNG, one PEARLS‐ED and one PEARLS‐CC course was conducted in 2023. In Vanuatu, one PEARLS‐ED course was conducted in 2022. Scan data were collected from the date of the respective course's completion until 06/09/2024, with varying index course dates.

### 2.1 Data Collection and Analysis

The data were conducted as an educational audit of the PEARLS program. No personal health information or identifiable participant data were collected, and participation in the education programme was voluntary. Participants were assigned nonidentifiable unique study IDs, and all ultrasound scans they uploaded to the Butterfly iQ cloud platform were reviewed by two independent researchers, who serve as data managers for the PEARLS program. These researchers manually counted the total number of scans and categorised them by ultrasound modality to assure data quality and reproducibility. Scans were transcribed into an Excel spreadsheet for analysis.

Under consensus by the research group, five types of ultrasound modalities that are most frequently used in emergency ultrasound were included for analysis: 1. Extended Focused Assessment with Sonography for Trauma (E‐FAST) 2. First‐trimester pregnancy ultrasound 3. Basic Critical Care Echocardiography (BCCE) 4. Lung ultrasound and 5. Other scans beyond the previous four modalities.

Scans were counted based on upload date and scan type, and no exclusions were made based on scan quality or completeness of minimum imaging sets. The total number of scans per participant and per category was calculated for each cohort, with a summary of the pooled results for the overall PEARLS cohort.

### 2.2 Statistical Analysis

Basic descriptive statistics were used to describe the PEARLS cohort, including mean and interquartile range (IQR) using Microsoft Excel statistical analysis package.

## 3 Results

The PEARLS‐ED courses encompassed a total of 40 participants across three cohorts from Vanuatu, Tonga and PNG (Tables 1 and 2). The PEARLS‐CC cohort consisted of 12 participants who followed on from the PNG PEARLS‐ED course (Table 3). The largest cohort of participants was PNG (*n* = 20). Both Vanuatu (*n* = 10) and Tonga (*n* = 10) had equal participants. In total, 576 scans were uploaded by the three study cohorts across the study period from 2022 to 2023. E‐FAST (*n* = 263) emerged as the predominant scan type across the entire cohort. This was followed by BCCE (*n* = 126) and Early Pregnancy (*n* = 81) modules (Table 4). Lung ultrasound was the scan modality the next most frequently performed (*n* = 56). Other scan types were the next most common (*n* = 53), consisting of gallbladder, ocular scans, late pregnancy scans and other various types that represent clinical use outside the PEARLS course curriculum. Only one participant in the PEARLS program so far has achieved completion of the program by uploading 25 scans in E‐FAST and First Trimester scanning.

**TABLE 1:** PEARLS‐ED attendee characteristics^a^.

		Number (total 40)	Percentage of total attendees
Seniority	Recent graduate	—	—
	Trainee/middle‐grade	22	55.0%
	Specialist	11	27.5%
	Other (including nondoctors)	1 (Sonography) 6 (Unknown)	17.5%
Specialty	ED	14	35.0%
	Rural generalist	5	12.5%
	General practice	2	5.0%
	ICU	2	5.0%
	Anaesthesia	2	5.0%
	Paediatric	1	2.5%
	O&G/surgical	6	15.0%
	Other	1 (Sonography) 1 (Radiology) 6 (Unknown)	20.0%

^a^ Pooled across initial 3 courses.

**TABLE 2:** Breakdown of PEARLS‐ED cohort participants in each cohort of learners.

	Vanuatu (ED) 2022	Tonga (ED) 2023	PNG (ED) 2023
Number of participants	10	10	20

**TABLE 3:** PEARLS‐CC attendee characteristics.

		Number (total 12)	Percentage of total attendees
Seniority	Recent graduate	—	—
	Trainee/middle‐grade	3	25%
	Specialist	8	67%
	Other (including nondoctors)	1	8%
Specialty	ED	2	17%
	Rural generalist		
	ICU	2	17%
	Anaesthesia	7	58%
	Paediatric	—	—
	O&G/surgical	—	—
	Other	1 (Health Extension Officer)	8%

**TABLE 4:** Scan modalities performed across the PEARLS program.

	Whole of program	E‐FAST	BCCE	First trimester	Lung	Other
Total	576	263	126	81	56	53
Percentage of total		45.7	21.9	14.1	9.7	9.2
Average	14.4	6.6	3.2	2	1.4	1.3
Median (IQR)	6.5 (12)	4 (9.5)	1 (4)	0 (1)	0 (2)	0 (1)

## 4 Discussion

The PEARLS project aims to deliver robust and sustainable POCUS education tailored to LMICs, in a highly clinically‐The data were conducted as an educational audit of the PEARLS program. No personal health information or identifiable participant data were collected, and participation was voluntary. This study was thus deemed exempt from formal ethics approval. Relevant manner specific to the emergency and critical care settings. This focus on capacity building in emergency and critical care has been identified by the World Health Assembly in a recent resolution as a health priority [11], with a global action plan to be developed over the next decade [12]. The PEARLS course has developed in an iterative manner responsive to learner demand. Our data show that the scan utilisation following a PEARLS course is variable, depending on the scan type and the setting. Future research will examine this phenomenon further. Thus far, the program has received highly positive feedback from attendees, and we look forward to building upon the course with advice and guidance from local experts (Appendix 3). There have been no issues encountered with training nonradiologists in POCUS, with all PEARLS programs supported by the hospital executives. Discussions regarding the development of future courses such as obstetrics, advanced echocardiography, musculoskeletal and more are ongoing. Our philosophy is to concentrate across the acute care spectrum, where the need for bedside imaging is most urgent. Our initial emphasis has been regional engagement with LMICs nearest to Australia and New Zealand, where most of our faculty are based. However, many similar groups to PEARLS exist globally, and we hope to collaborate and partner with others as our reach extends further afield.

PEARLS participants who demonstrate teaching and leadership skills, enthusiasm, and competence based on the quality of their postcourse cloud uploads and engagement with the longitudinal learning, are selected for inclusion in a faculty development program. This can be delivered in a hybrid manner, remotely before a return PEARLS visit, and then in‐person on a day before delivered courses. These learners hereby graduate to become PEARLS ‘apprentice faculty’ on the return PEARLS visit and can more effectively drive ongoing learner engagement due to being embedded in‐country. Ideally, over time, reliance on visiting faculty will progressively decrease over successive years.

Accurately assessing the efficacy of the project is important as the volume of our courses increases. Relevant indicators to assess will include a percentage of participants engaging with online review, number of participants meeting competency requirements over a year, ongoing review of common barriers to engagement (e.g., uploading studies), timing of image review by coaches, adequacy of imaging, etc. Learners are requested to provide informed consent to participate in research at the start of each PEARLS course.

## 5 Challenges

### 5.1 Precourse Planning and Learner Preparation

Learner selection is critical to maximise the probability of ongoing postcourse engagement and educational progression. A combination of self‐selection and selection by local leaders identifies those with the capacity to be engaged as required for both the course and the longitudinal component. The high clinical workload and limited nonclinical time are significant barriers for clinicians to incorporate POCUS into their workflow in many of the LMICs where PEARLS has been run. However, even course attendance can, at a minimum, help these clinicians to understand the new skillset of POCUS being employed within their health systems. Additional work is required to systematically identify which learners have the highest likelihood of maintaining postcourse engagement, as well as which craft groups are most likely to benefit from the integration of POCUS into their clinical workflow.

Completion of the precourse learning is vital to obtain the most benefit from course attendance. The in‐person course lectures are designed to consolidate precourse content, so learners who attend the course unprepared receive a less comprehensive educational package. Reasons for incomplete precourse learning are diverse, but may include difficulties communicating with learners, courses being planned at short notice and poor access to a stable internet connection in many areas.

Course planning and timing requires close collaboration with local leaders. Avoiding clashes with other events is important, but a course can be scheduled opportunistically before or after regional physicians convene for another purpose, for example, an annual rural generalists' conference.

### 5.2 Course Delivery

Funding is sought for flights, accommodation, equipment and course venue hire through various avenues, but some degree of self‐funding by faculty and learners has been required at times. Funding sources are acknowledged in the declarations section of this paper.

Course content is intended to be individualised to local needs, which requires significant lead‐in time for consultation and preparation of requested content. The division of learners and content between the PEARLS‐ED and PEARLS‐CC courses has been difficult over many courses, with learners from all clinical backgrounds being enthusiastic to attend both courses. Examples of this cross‐specialty interest include intensive care doctors who might appreciate the immediacy of identifying an intrauterine pregnancy taught on the PEARLS‐ED course, or a rural doctor seeing the benefit of echocardiography in a critically ill patient taught on the PEARLS‐CC course.

The OSCE assessment is intended to be dual purpose: the obvious element of assessing image acquisition and interpretation skills, but also assessing learner ability to complete the technical steps of uploading a completed study to the online cloud for image review. We see this as an important confirmation that learners will be able to continue engaging postcourse. However, unstable internet connections at the course venue, time constraints, login glitches and necessity for probe sharing mean the latter element has not always been achievable.

All learners attending so far have been fluent English speakers, as the countries visited all have had English as one of their official languages, but language barriers are anticipated in future courses. Cultural safety and cultural competency are identified as faculty requirements, with many of the faculty having diverse experience working in LMICs. Gender equity and social inclusion are actively considered in the course planning, but are dependent on many of the host countries for engagement in this process.

### 5.3 Remote Mentorship, Image Interpretation and Integration Into Clinical Practice

The most significant barrier to image uploading has been limited access to a stable internet connection or phone data in Pacific Island nations, particularly in more rural settings. Unfortunately, these isolated doctors are the very ones who will benefit most from remote feedback. PEARLS is not a credentialing process regardless of the completion of the competency pathway described above, and clinicians involved are encouraged and supported to explore this process within the context of their own healthcare system.

Probe damage and maintenance is also a concern that is anticipated. The ultrasound probes are covered by at least a 1‐year manufacturer warranty, with replacement as an option on a case‐by‐case basis. Some machines' warranties are extended to 2–3 years. Beyond this timeframe, it would become the responsibility of the clinician or hospital. PEARLS Country Lead and Tech Support Team will help liaise clinicians through this process. So far, one probe has been replaced secondary to external damage. Probes that are unused by the learners may be redistributed to a peer following a discussion with the learner regarding barriers to use and uploading, as per the PEARLS agreement signed by the participants before the course.

Feedback that is provided promptly has the highest potential for educational impact, as details of a particular case will still be fresh in a learner's mind. Additionally, although PEARLS is a primarily educational project rather than a remote management advice service, feedback needs to be rapid to have any possibility of influencing urgent management decisions. When competing demands on a particular PEARLS Coach result in delayed feedback, their faculty peers can provide substitute reviews. It has been identified through our longitudinal ‘coaching’ process that many of the clinicians interested and able to effectively teach POCUS in a short course format may not have the skill set needed for remote review and feedback of imaging, and thus a ‘coach mentor’ system has allowed faculty development of this skill set.

## 6 Limitations

There are some limitations to consider. Firstly, this study only describes the amount of ultrasound scans uploaded to the Butterfly iQ platform and does not account for the total number of scans performed by participants. Informal follow‐up indicates that participants perform scans without saving or uploading images, and the data likely underrepresent the scanning practices of participants in the PEARLS program.

## 7 Conclusion

The PEARLS program aims to provide robust POCUS education in LMICs utilising novel elements to allow ongoing learner progression following an initial POCUS short course. Sustainability and penetration of POCUS training is an elusive goal in the ultrasound educational community. Future research must include an examination of the barriers and enablers to scan uptake and utilisation, as well as attainment of proficiency. Further data are required to assess the impact on learner and patient outcomes. We hope the bundle of cloud‐based image review, pairing learners with their POCUS coach, provision of regular lectures and image review sessions provides the best available platform for learner growth.

## Author Contributions

B.H., L.M. and J.H. conceptualised the article. W.C.T. and I.C. supported data curation and data analysis. V.A., S.O., J.H. and L.M. provided expert advice in writing as well as editing. V.A., I.J.B. and A.K. provided expert review and in‐country insights, a major contribution to the overall manuscript. All authors read, edited and approved the final manuscript.

## Ethics Statement

This was an educational audit of the PEARLS program. This study was thus deemed exempt from formal ethics approval. Anonymised participant comments are included with permission.

## Consent

All PEARLS course attendees who provided feedback to the PEARLS program that is being used for this article provided written consent for their comments and feedback to be used for publication.

## Conflicts of Interest

The authors declare no conflicts of interest.

## Data Availability

Data sharing is not applicable to this article as no datasets were generated or analysed during the current study.

## Appendix 3

Surveys conducted at the end of the course contained the following comments:

**Table:**

PEARLS‐ED Vanuatu 2022	PEARLS‐ED & CC PNG 2023
*‘Very useful and applicable to our setting’* *‘Extremely relevant in low‐resource setting’* *‘Very honoured & grateful for the opportunity’*	*‘Thank you for this opportunity. Working in a provincial hospital that has limited resources, including staff, it is difficult to get the right people to do timely echoes’*
*‘Has been a wonderful experience to date. Really happy to have been part of the program’* *‘Thoroughly enjoyed this course. Extremely practical!’*	*‘An excellent course that will help make clinical diagnosis to assist us with patient management. I recommend this course to be taught to all doctors who take care of critically ill patients’*
*‘Forever grateful for having the opportunity to attend this workshop and learn from excellent & friendly instructors. Excellent platform (cloud) to grow and develop skills tomorrow onwards’*	*‘There has never been any form of ultrasound or echo taught by undergrad and postgrad medical education in PNG apart from those in radiology. The content and structure of how it was taught in theory and practically is a first time for most participants, and all have understood well and can apply practically in their respective centres’*
